# Role of baseline echocardiography prior to initiation of anthracycline-based chemotherapy in breast cancer patients

**DOI:** 10.1186/s12885-014-1004-0

**Published:** 2015-01-21

**Authors:** Alain Mina, Hind Rafei, Maya Khalil, Yasmine Hassoun, Zeina Nasser, Arafat Tfayli

**Affiliations:** American University of Beirut, Beirut, Lebanon; Department of Internal Medicine, Internal Medicine Resident-University of Miami Miller School of Medicine-Palm Beach Regional Campus, Miami, USA; Department of Internal Medicine, American University of Beirut Medical Center, Beirut, Lebanon; Faculty of Public Health, Free University of Brussels, Brussels, Belgium

**Keywords:** Breast cancer, Echocardiography, Anthracycline

## Abstract

**Background:**

Anthracycline adjuvant therapy has taken a particular role in the treatment of early stage breast cancer with an associated decrease in rates of both relapse and death. Their success however has been limited by their myelosuppression and their well-established risk of cardiac dysfunction. Guidelines have emerged that would limit the maximum lifetime dose of anthracyclines and make a baseline assessment and periodic monitoring of cardiac function part of the routine practice, which could be cumbersome, and may condemn the patient to an unwarranted modification of his/her regimen. Our study aimed at assessing the incidence of abnormal baseline echocardiography in asymptomatic women with breast cancer prior to anthracycline therapy and establishing risk criteria associated with abnormal echocardiograms at baseline.

**Methods:**

220 Patients seen at AUBMC (American University of Beirut Medical Center) who had non- metastatic breast cancer, and had an echocardiography performed before starting anthracycline chemotherapy were chosen. Data about demographic characteristics, tumor characteristics, baseline echocardiography results, and change in clinical decision was collected. Patients with suboptimal (less than 50%) ejection fraction (EF) on baseline echocardiography were analyzed for the prevalence of cardiac risk factors. Results were compared to those among the overall study group using Fisher’s Exact test. A p- value of = < 0.05 was used as reference for statistical significance.

**Results:**

All 220 of our patients had received a baseline echo prior to initiation of anthracycline therapy. 6.7% of these patients had already some abnormality in wall motion but only 2.7% had a suboptimal ejection fraction. 1.3% had a change in chemotherapy regimen based on ejection fraction. The patients with depressed EF had higher rates of CAD (coronary artery disease), diabetes, hypertension and dyslipidemia than the overall study group but without statistical significance.

**Conclusions:**

Our study, as well as the previous contingent studies raise the question about routine echocardiography prior to anthracycline therapy and might eventually lead to a modification of current practice guidelines.

## Background

Over the last few decades, the arsenal of chemotherapeutic agents available to oncologists, and specifically breast cancer specialists has been constantly expanding. The use of anthracyclines has become particularly universal and central to every accepted treatment algorithm. Anthracycline adjuvant therapy has taken a particular role in the treatment of early stage breast cancer with an associated decrease in rates of both relapse and death [1,2].

Commonly used anthracyclines in adjuvant regimens however, such as Adriamycin and Epirubicin, are well known for their cardiotoxicity that increases with factors such as dosage, older age, use of other cardiotoxic agents (such as trastuzumab), emphysema, diabetes and an underlying cardiac disease [2-4]. Given their indispensable role, an intervention aimed at controlling their ill-effects was a must. For instance, guidelines have emerged that would limit the maximum lifetime dose of doxorubicin to a range of 450 mg/m^2^ to 550 mg/m^2.^ [5,6]. Furthermore, a baseline assessment and periodic monitoring of cardiac function (left ventricular ejection fraction and wall motion abnormality) have been the routine practice with any patient receiving cardiotoxic chemotherapeutic agent regimens as per the recommendation of the American College of Cardiology/American Heart Association.

These precautionary practices, although at the time justified with the prevailing data, are quite costly and cumbersome, not only entailing additional visits, procedures, and costs but also condemning the patient to an unwarranted modification of his/her regimen that may or may not be in his/her best interest. Many studies have shown that it is in very few instances that physicians have had to alter the anthracycline dosage, or regimen itself, based on echocardiographic evidence of a decreased left ventricular function [3,5]. Furthermore, the wide majority of previously healthy patients have been shown to have consistently normal cardiac function at baseline and on echocardiography follow-ups, despite completing the recommended anthracycline course [3,5,7]. And so the need for baseline echocardiography and periodic monitoring of left ventricular function in previously healthy and asymptomatic patients receiving anthracyclines becomes debatable, especially that a clear-cut cost effectiveness has not been established, nor do chemotherapeutic protocols reach the toxic cumulative maximal dose [6].

Practice at our center entails that all patients receiving anthracycline as part of their chemotherapy protocol undergo baseline investigative echocardiography. Screening is not only costly and cumbersome as mentioned earlier but is far from flawless. The sensitivity and specificity of Echocardiography for the detection of Left ventricular function are 80% and 88% respectively with a positive predictive value of 86% [8] and so false positives or negatives though unlikely, are possible, further adding to the hazard of an unwarranted change in chemo regimen or dosage. Our current study aims at assessing the incidence of an abnormal echocardiography result performed on asymptomatic women with breast cancer prior to anthracycline therapy and establish risk criteria associated with abnormal echocardiograms at baseline. The results of our data analysis will depict a pattern of echocardiography impact on the physician’s choice of chemotherapy regimen as well as weigh the need for a baseline echocardiography in our population of breast cancer patients. Our results are bound to have implications on future management and practice.

## Methods

Patients seen at our center that had a diagnosis of breast cancer between 2004 and 2012,underwent an echocardiography before starting the first chemotherapy session and meeting the inclusion ciriteria, were the subjects of this analysis and were chosen consecutively. Those with metastatic disease (Stage IV disease) were excluded from the study. Exclusion criteria also included patients who didn’t receive anthracyclines irrespective of their cardiac status, as well as those with a primary breast lymphoma or phyllodes tumor.

This study was approved by the ethics committee of the American University of Beirut International Review Board and a consent form was waived.

The study followed a cross-sectional design. Patients’ records were reviewed in detail. Data collection included demographic characteristics of patients, tumor characteristics (stage, grade, TNM classification, laterality, histology, hormonal status…), medical history (dyslipidemia (defined as LDL > 130, Triglycerides >150 or HDL <40), diabetes (defined as a fasting plasma glucose of > 126), hypertension (defined as a blood pressure superior to140 mmHg systolic or 90 mmHg diastolic, coronary artery disease (atherosclerotic cardiovascular disease diagnosed by baseline electrocardiography (ECG), stress ECG, echocardiography or coronary angiography), signs/symptoms of cardiac disease (chest pain, cough, orthopnea etc.), smoking, menopausal status…), baseline echocardiography results, clinical decision (chemotherapy regimen, change in chemotherapy based on echocardiography results, previous chemotherapy, previous hormonal therapy, surgery…). The echocardiography was performed at AUBMC, in the noninvasive cardiac lab. Different measurement apparatuses were used over the years and left ventricular ejection fraction was assessed based on 2D measurements.

Data entry was performed by independent lay persons that were unaware of the objectives of the study. Data cleaning was carried out by the researchers. Data analysis was performed using SPSS IBM version 20.0 and initially aimed at identifying patients with an abnormal echocardiography results, suboptimal ejection fraction and those with a change in chemotherapy regimen based on echocardiography results. Patients who turned out to have a suboptimal ejection fraction at baseline echocardiography were analyzed for the prevalence of dyslipidemia, hypertension, CAD, diabetes, smoking, and morbid obesity. Results were compared to those among the overall study group using Fisher’s exact test. A p- value of = < 0.05 was used as reference for statistical significance. A multivariate analysis using logistic regression was performed after ensuring the adequacy of our data using the Hosmer and Lemeshow test, to detect any association between EF at baseline and cardiovascular risk factors (Baseline Ejection Fraction was the dependent variable). Adjusted odds ratios and their 95% confidence intervals were reported.

## Results

A total of 220 patients met the study criteria and their records were reviewed in detail. Table 1 summarizes patient characteristics. Essentially 98.6% were females with a mean age of 51.5 years. This relatively young mean age reflects the Lebanese breast cancer population whose mean age at presentation has been reported at 50 [9]. Forty nine percent of patients were post-menopausal. Around 68% of patients had stage I or II, and 45% had a grade 3 tumor. Majority of tumors were ER/PR positive (71.8% ER positive and 63.6% PR positive) and 23.6% were her-2 overexpressing.

**Table 1:** **Demographics and tumor characteristics**

**Variables**	**Groups**	**N**	**Percentages**
Stage	1	47	21.4
	2	102	46.4
	3	57	25.9
	Not available	14	6.3
Grade	1	29	13.2
	2	80	36.4
	3	98	44.5
	Not available	13	5.9
Laterality	Right side	110	50
	Left side	109	49.5
	Bilateral	1	0.5
T	1	74	33.6
	2	97	44.1
	3	37	16.8
	Not available	12	5.5
N	0	92	41.8
	1	82	37.3
	2	23	10.4
	3	18	8.2
	Not available	5	2.3
M	0	220	100
^a^ER	Negative	60	27.3
	Positive	158	71.8
	Not available	2	0.9
^b^PR	Negative	78	35.5
	Positive	140	63.6
	Not available	2	0.9
^c^HER-2	Negative	118	53.6
	Equivocal	67	30.5
	Overexpressed	52	23.6
	Not available	3	1.36
Previous hormone therapy (PHT)	No PHT	131	59.6
	Has had PHT	43	19.5
	Not available	46	20.9
Menopausal status	Premenopausal	90	40.9
	Postmenopausal	109	49.5
	Not available	21	9.6

^a^ER: Estrogen Receptor Status. ^b^PR: Progesterone Receptor Status. ^c^HER2: HER-2 Receptor Status.

As per the inclusion criteria, all 220 of our patients received a baseline echocardiogram prior to initiation of anthracycline therapy. Fifteen (6.8%) of these patients had some abnormality in wall motion but only 6 (2.7%) had a suboptimal ejection fraction (less than 50%) and 3 of those, i.e. 1.35% had a change in chemotherapy regimen based on ejection fraction reading.

We reviewed the records of all of our patient population for prevalence of cardiac disease risk factors (Table 2). In Table 3, we compared the distribution of cardiac risk factors among patients with abnormal echo to those with normal echo, to see if it were these risk factors that would explain the abnormal LVEF. Two of the newly isolated group had a prior diagnosis of coronary artery disease, two patients had diabetes, none of the patients was a smoker, one patient was morbidly obese, and two patients had dyslipidemia as compared to 2.3% of the overall study group with coronary artery disease, 16% with diabetes, 24.3% with hypertension, 34.6% smokers and 31.8% obese (BMI > 30) and so these risk factors were more prevalent in the 6 patients that demonstrated a clinically significant abnormal baseline echocardiography as compared to the patients with normal echocardiography. However, these differences were not statistically significant (Table 3). A multivariate logistic regression was performed as shown in Tables 4 and 5, and Coronary Artery Disease with an odds ratio of 4.2, 95% CI (2.1-15.4) was the only risk factor that showed significant association with a suboptimal baseline ejection fraction.

**Table 2:** **Study group risk factors for cardiac disease**

**Variables**	**Groups**	**N**	**Percentages**
Dyslipidemia	No	170	77.2
	Yes	40	18.2
	Not available	10	4.6
Hypertension	No	155	70.5
	Yes	55	25
	Not available	10	4.5
^a^CAD	No	203	92.3
	Yes	7	3.2
	Not available	10	4.5
Diabetes	No	175	79.5
	Yes	36	16.4
	Not available	9	4.1
Smoking	No	131	59.5
	Yes	74	33.6
	Not available	15	6.9

^a^CAD: Coronary artery disease.

**Table 3:** **Distribution of cardiac risk factors among patients with abnormal echo compared to those with normal echo - Fisher’s Exact test**

**Variable**	**Normal echo**	**Abnormal echo**	**P value**
Diabetes			0.3
No	171 (79.9)	4 (66.7)	
Yes	34 (15.9)	2 (33.3)	
Not available	9 (4.2)		
Dyslipidemia			0.3
No	166 (77.7)	4 (66.7)	
Yes	38 (17.6)	2 (33.3)	
Not available	10 (4.7)		
Hypertension			0.2
No	152 (71)	3 (50)	
Yes	52 (24.3)	3 (50)	
Not available	10 (4.7)		
^a^CAD			0.01
No	199 (93)	4 (66.7)	
Yes	5 (2.3)	2 (33.3)	
Not available	10 (4.7)		
Smoking status			0.1
No	126 (58.9)	5 (83.3)	
Yes	74 (34.6)	0 (0)	
Not available	14 (6.5)	1(16.7)	
^b^BMI			1
<25	55 (25.7)	2 (33.3)	
25-30	74 (34.6)	2 (33.3)	
>30	68 (31.8)	1 (16.7)	
Not available	17 (7.9)	1 (16.7)	

^a^CAD: Coronary artery disease ^b^BMI: Body Mass Index.

**Table 4:** **Association between suboptimal EF at baseline and risk factors**

**Variable**	**Non**	**Yes**	**P value**
Diabetes			0.3
Non	167 (83.1)	4 (66.7)	
Yes	34 (16.9)	2 (33.3)	
Dyslipidemia			0.3
No	163 (81.5)	4 (66.7)	
Yes	37 (18.5)	2 (33.3)	
Hypertension			0.2
No	149 (74.1)	3 (50.0)	
Yes	52 (25.9)	3 (50.0)	
^a^CAD			0.01*
No	195 (97.5)	4 (66.7)	
Yes	5 (2.5)	2 (33.3)	
Smoking status			0.1
No	124 (63.3)	5 (100.0)	
Yes	72 (36.7)	0 (0.0)	
^b^BMI			
0 (18–25)	54 (28.0)	2 (40)	1.0
1 (25–30)	72 (37.3)	2 (40)	
2 (30–35)	46 (23.8)	1 (20)	
3 (35–40)	15 (7.8)	0 (0.0)	
4 (>40)	5 (2.6)	0 (0.0)	
5 (<18)	1 (0.5)	0 (0.0)	

*P value < 0.05 statistically significant. ^a^CAD: Coronary artery disease. ^b^BMI: Body Mass Index.

**Table 5:** **Adjusted odds ratio with 95% confidence interval for the logistic regression**

**Variable**	^**b**^ **OR**	**95** ^**c**^ **CI**	**P value**
^a^CAD	4.2	2.1 -- 15.4	0.01*

^a^CAD: Coronary artery disease, ^b^OR: adjusted odds ratio, ^c^CI: confidence interval, *P value <0.05 statistically significant.

## Discussion

Anthracycline cardiotoxicity has been well established in the medical literature and its mechanisms well elucidated. With their growing role in the management of neoplastic diseases, particularly that of breast cancer, precautionary measures such as baseline investigative echocardiography has been the standard protocol in all breast cancer patients being treated with an anthracycline. Furthermore, a thorough and global assessment of the clinical condition of the patient has become a must as the cardiotoxicity rate associated with the commonly used regimens (such as Adriamycin and Epirubicin) was found to increase not only in a dose-dependent fashion (cumulative dose and dose per treatment) but also with various unrelated factors such as other cardiotoxic drugs (e.g. Trastuzumab), diabetes mellitus, the rate of administration of the drug, a preexisting heart disease (whether ischemic in nature or a decrease in the baseline ejection fraction), previous mediastinal irradiation, age, sex (female) etc. [10].

A look into our sample for the prevalence of such factors, particularly those that are predictors of cardiac disease, revealed that among the six patients who had abnormal baseline echocardiography, two had coronary artery disease (33.3%), 2 had dyslipidemia (33.3%), 2 had diabetes (33.3%), None were smokers (0%), 1 was morbidly obese (16.7%) and 3 were hypertensive (50%). The prevalence of these comorbid conditions in these patients is more than that of patients with normal echocardiography, however with no statistical significance. Given the relatively small number of patients with abnormal baseline EF, the association between risk factors of cardiac disease and the presence of an abnormal echocardiography at baseline cannot be established nor denied. A multivariate logistic regression (Table 4) only showed a statistically significant association between baseline EF and Coronary Artery Disease. Perhaps a larger sample would have decreed more solid and reliable associations. An additional limitation could have been that our sample was extracted from a single medical center, and our protocols, practices and patient population does not necessarily reflect those of all other reputable medical centers.

## Conclusions

Out of 220 of our patients, only 6 had abnormal baseline echocardiograms (2.7%), of which only 3 (1.35%) had a change in chemotherapy regimen based on EF reading. Hence, the impact of the baseline echocardiography on the managerial decision of our oncologists has been minute. Also the association of irreversible cardiac myocyte damage and long term sequelae with continuation of treatment (within acceptable maximal doses) has not been well exposed. Add to that the fact that screening with echocardiography is far from flawless with a sensitivity and specificity of 80% and 88% respectively [8], giving room for false positives and negatives, as well as the hazard of an unnecessary change in chemotherapy regimen or dosage. All this is added to the psychological and economic burdens such information might carry over the patient’s well-being. With that in mind, our study as well as previous contingent studies, raises the question about routine echocardiography prior to anthracycline therapy and might eventually lead to a modification of current practice guidelines. Furthermore, particular attention should be made to measures known to limit anthracycline-associated cardiotoxicity such as limiting the cumulative dose of doxorubicin to a range of 450 mg/m^2^ to 550 mg/m^2^ [5,6], infusing the regimen slowly, coadministration of a protective agent (dexrazoxane) when reaching high doses of anthracyclines and potentially using the less cardiotoxic liposomal preparations [10]. The benefit of the physician’s attentiveness and awareness of these clinical elements and precautions, could outweigh that of the baseline echocardiography and avoid its cumbersome and hazardous implications on both the patients’ management and well-being.
